# The Performance of GeneXpert in the Diagnosis of Lymph Node Tuberculosis: A Prospective Study Comparing GeneXpert and Culture Findings

**DOI:** 10.7759/cureus.64979

**Published:** 2024-07-20

**Authors:** Mohamed Afellah, Sofia Zoukal, Najib Benmansour, Abdelilah Arioua, Naouar Ouattassi, Mohamed Noureddine El Amine El Alami

**Affiliations:** 1 Otolaryngology, Hassan II University Hospital, Fez, MAR; 2 Laboratory of Epidemiology, Faculty of Medicine and Pharmacy/University Hassan II, Casablanca, MAR; 3 Otolaryngology - Head and Neck Surgery, Hassan II University Hospital, Fez, MAR

**Keywords:** spicemen, performance, genexpert, lymph node, extra pulmonary tuberculosis

## Abstract

Background and objective

Lymph node tuberculosis (LNTB) is a common manifestation of extrapulmonary tuberculosis (EPTB). GeneXpert is a rapid diagnostic molecular test that simultaneously detects tuberculosis and rifampicin (RIF) resistance. In this study, we aimed to assess the epidemiology of LNTB and diagnostic performance parameters of the GeneXpert in routine ENT practice.

Methods

We conducted a cross-sectional prospective study from January to July 2019, in the Department of Otorhinolaryngology and Head Neck Surgery at the Hassan II University Hospital Center of Fez, Morocco. The samples were collected using lymph node biopsy and subjected to GeneXpert assay, culture, and histopathology. Diagnostic performance parameters of the GeneXpert were calculated and compared with culture.

Results

All patients with cervical adenopathy were included. Lymph node biopsies were performed for all patients. The performance of the GeneXpert was assessed according to culture findings. Among the 75 cases, the mean age was 21.6 ± 12.7 years with a female predominance (60%). GeneXpert was positive in 66.7% of specimens. The sensitivity and specificity of the GeneXpert assay were 78.6% and 40.4% respectively. GeneXpert accuracy was 54.6%. The positive predictive value (PPV) and negative predictive value (NPV) were found to be 44% (95% CI: 30.2-57.8) and 76% (95% CI: 59.3-92.7) respectively. Mycobacterium bovis was isolated in all samples, with no case of resistance to RIF found.

Conclusions

The performance of GeneXpert was found to be superior in terms of establishing the diagnosis of LNTB. It offers speedy and prompt results and clinicians should adopt it in routine clinical practice.

## Introduction

Tuberculosis (TB) is a global public health concern. According to the World Health Organization (WHO), it led to 1.5 million deaths in 2020, with 10 million new cases reported [1]. The disease usually affects the lungs (pulmonary TB), but can also affect other sites [extrapulmonary TB (EPTB)]. Lymph node tuberculosis (LNTB) is a common manifestation of all EPTB cases. It is often located in the cervical lymph nodes and is one of the leading health issues in the developing world [2].

LNTB was known to be non-contagious but has become a growing problem. Patients usually present with cervical adenopathy associated with sparse systemic symptoms, and diagnosis can be difficult and delayed [3]. Also, the detection of Mycobacterium tuberculosis (MTB) by culturing, the best reference standard, takes six to eight weeks and requires invasive surgical procedures that are traumatic for patients. It may also lead to misclassification owing to the paucibacillary nature of the disease [4]. In 2010, a rapid molecular test (GeneXpert) was endorsed by the WHO, and the identification of MTB through GeneXpert has been shown to be rapid [5,6]. It is currently recommended as the first-line assay for simultaneously diagnosing EPTB and rifampicin (RIF) resistance, especially in HIV-positive individuals and children who cannot spit [7]. Recently, a meta-analysis has shown that GeneXpert is very effective for the diagnosis of LNTB compared to culture. The pooled sensitivity and specificity were 84% (95% CI: 77-90%) and 91% (95% CI: 78-96%) respectively [8].

In Morocco, where TB incidence in 2020 was 98 cases for 100,000 inhabitants nationwide [9] and 79/100,000 in cities like Fez [10], the overall treatment success rate was 80%, and the loss-to-follow-up (non-adherence) rate was as high as 11% [11]. It remains the most common infectious cause of death after neonatal disorders and low respiratory infections, with a rate of 37% for LNTB [12]. Despite the success in controlling TB, the proportion of EPTB increased from 28% to 48% in 2019 among all newly notified cases [13]. As per the National TB Program, the diagnosis of LNTB should be based on the clinical picture, including histopathological evidence. However, histopathology is highly sensitive but not very specific, whereas culture is rarely used to confirm a diagnosis [14]. In light of this, this study aimed to assess the performance of GeneXpert MTB/RIF in the diagnosis of LNTB and RIF resistance after seven months of acquisition in a small prospective cohort at Fez.

## Materials and methods

Study design and population

The prospective observational study was conducted per the STARD (Standards for Reporting Diagnostic Accuracy) guidelines [15] in the Department of Otorhinolaryngology and Head Neck Surgery (ENT) of Omar Drissi Hospital, a tertiary care center of the University Hospital Center (Fez, Morocco). It was performed between January 2019 and July 2019, and the cohort was drawn from the national EPTB surveillance registry. Patients of all age groups willing to be part of the study and admitted to ENT for cervical adenopathy suggesting LNTB were included. All cases were interviewed using a standardized questionnaire, which included the sociodemographic data (age, gender, residence, education level), TB history, milk consumption, clinical characteristics, laboratory results (intradermal tuberculin reaction, bacteriological and histopathological examination), and susceptibility to RIF.

Sample collection

Lymph node biopsies indicating LNTB were performed for all patients during surgical cervicotomy. They were aseptically collected, frozen at −20°C, and transported in cooling boxes containing ice packs to the Ibn Nafis laboratory at Fez, where they were analyzed. Lymph node specimens were tested with direct smear microscopy, solid and liquid culture, and the new molecular-based GeneXpert to provide the bacteriological evidence. It was performed according to the standard protocol recommended by the WHO [16].

Case definition

Patients were categorized based on histological and microbiological results. Typical histopathology finding for LNTB is the presence of caseous necrosis in the inflammatory granuloma. A bacteriological examination was considered positive when a culture was positive in a lymph node biopsy [17]. Phenotypic identification was based on the niacin test, nitrate reduction test, susceptibility to thiophene-2-carboxylic acid hydrazide, and growth on p-nitrobenzoic acid medium [18].

Statistical analysis

Qualitative variables were presented as numbers and frequencies. Quantitative variables were presented as mean ± standard deviation (SD) (normal distribution) or as median with interquartile range (IQR). The chi-square test or Fisher's exact test was used to compare differences between categorical variables. The performance of GeneXpert was calculated using the culture as a reference and expressed as sensitivity, specificity, positive predictive value (PPV), and negative predictive value (NPV). A p-value <0.05 was considered statistically significant. All statistical analyses were performed with R software version 4.0.5.

Ethical considerations

Written informed consent was obtained from all patients included. This study was approved by the Ethics Committee for Biomedical Research (CERB) of the Faculty of Medicine and Pharmacy of Rabat (approval number: 84/16). It was carried out by adhering to the relevant guidelines and regulations.

## Results

Patient characteristics

This study included 75 cases of cervical lymphadenopathy. The mean age of the patients was 21.6 ± 12.7 years, and the cohort had a female predominance (60%). A total of 66 patients (88.0%) lived in urban areas. Twenty-six patients were found to be HIV-negative, and 8% of the patients (n=6) had been previously treated for tuberculosis. The predominant milk consumption was Lben (92.4%, n=61). The documented presenting symptoms were weight loss (62.7%, n=47) and fever (60.0%, n=45 ). The demographic and baseline characteristics of the study participants are summarized in Table 1.

**Table 1:** Demographic and baseline characteristics of the participants (N=75) *P<0.05

Variables	Total, n (%)	Lymph node tuberculosis		P-value
		Yes, n (%) (n=28)	No, n (%) (n=47)	
Sex				0.922
Female	45 (60.0)	17 (37.8)	28 (62.2)	
Male	30 (40.0)	11 (36.7)	19 (63.3)	
Age, years				0.751
˂15	23 (30.7)	9 (39.1)	14 (60.9)	
15-30	36 (48.0)	11 (30.6)	25 (69.4)	
30-45	10 (13.3)	6 (60.0)	4 (40.0)	
≥45	6 (8.0)	2 (33.3)	4 (66.7)	
Residence				1.000
Urban	66 (88.0)	25 (37.9)	41 (62.1)	
Rural	9 (12.0)	3 (33.3)	6 (66.7)	
Educational level				0.399
Illiterate	25 (33.3)	11 (44.0)	14 (56.0)	
Educated	50 (66.7)	17 (34.0)	33 (66.0)	
Income				0.911
Stable	2 (2.7)	1 (50.0)	1 (50.0)	
Not regular	20 (26.7)	7 (35.0)	13 (65.0)	
No income	53 (70.7)	20 (37.7)	33 (62.3)	
Tuberculosis contagion	5 (6.7)	3 (60.0)	2 (40.0)	0.356
Previously treated with antituberculosis medication	6 (8.0)	1 (16.7)	5 (83.3)	0.401
Diabetes	1 (1.3)	1 (100.0)	0 (0.0)	0.373
HIV status				0.723
Negative	26 (34.7)	9 (34.6)	17 (65.4)	
Unknown	49 (65.3)	19 (38.8)	30 (61.2)	
Smoking	5 (6.7)	3 (60.0)	2 (40.0)	0.356
Milk consumption				
Farm milk	40 (60.6)	16 (40.0)	24 (60.0)	0.659
Jben	32 (49.2)	14 (43.8)	18 (56.2)	0.261
Lben	61 (92.4)	24 (39.3)	37 (60.7)	0.642
Symptoms at the time of inclusion				
Fever	45 (60.0)	22 (48.9)	23 (51.1)	0.011*
Weightloss	47 (62.7)	21 (44.7)	26 (55.3)	0.088
Intradermal tuberculin reaction				0.571
Yes	59 (78.7)	23 (39.0)	36 (61.0)	
No	16 (21.3)	5 (31.3)	11 (68.8)	

Diagnostic accuracy of GeneXpert

The median duration from first admission to molecular test results was one day (IQR: 0-2), while the median time to histopathological results was 10 days (IQR: 4-8) and the median time to culture results was seven weeks (IQR: 6-8). Histopathology was positive for 69.3% of specimens and showed granulomatosis inflammation, with necrosis observed in 52 cases. The positivity rate for GeneXpert was 66.7% (n=50) while that for culture was 37.3% (n=28) (Table 2).

**Table 2:** Histopathological, GeneXpert, and Culture findings among the participants (N=75)

Results	N (%)
Histopathology	
Granulomatosis inflammation with necrosis	52 (69.3)
Granulomatosis inflammation without necrosis	9 (12.0)
Suppurative inflammation	9 (12.0)
Hodgkin's lymphoma	5 (6.6)
GeneXpert	
Positive	50 (66.7)
Negative	25 (33.3)
Culture	
Positive	28 (37.3)
Negative	47 (62.7)

The sensitivity and specificity of GeneXpert assay in comparison to culture were 78.6% (95% CI: 63.4-93.8) and 40.4% (95% CI: 26.4-54.5). PPV was 44% (95% CI: 30.2-57.8), whereas NPV was 76% (95% CI: 59.3-92.7). The accuracy of GeneXpert in the diagnosis of LNTB was 54.6% (Table 3).

**Table 3:** GeneXpert accuracy according to culture findings among the participants (N=75) CI: confidence interval; LNTB: lymph node tuberculosis; NPV: negative predictive value; PPV: positive predictive value

	Culture		Sensitivity % (95% CI)	Specificity % (95% CI)	PPV % (95% CI)	NPV % (95% CI)	P-value
	LNTB+	LNTB-					
Xpert+	22	28	78.6 (63.4-93.8)	40.4 (26.4-54.5)	44.0 (30.2-57.8)	76.0 (59.3-92.7)	0.401
Xpert-	6	19					

The sensitivity and specificity of GeneXpert assay in comparison to histopathology were 75.0% (95% CI: 63.2-86.8) and 52.2% (95% CI: 31.7-72.6). PPV was 78.0% (95% CI: 66.5-89.5), whereas NPV was 48.0% (95% CI: 28.4-67.6). The accuracy of GeneXpert in the diagnosis of lymph node tuberculosis was 68.0%, which was statistically significant as evidenced by a p-value of 0.021 (Table 4).

**Table 4:** GeneXpert accuracy according to histopathology findings among the participants (N=75) *P<0.05 CI: confidence interval; LNTB: lymph node tuberculosis; NPV: negative predictive value; PPV: positive predictive value

	Histopathology		Sensitivity % (95% CI)	Specificity % (95% CI)	PPV % (95% CI)	NPV % (95% CI)	P-value
	LNTB+	LNTB-					
Xpert+	39	11	75.0 (63.2-86.8)	52.2 (31.7-72.6)	78.0 (66.5-89.5)	48.0 (28.4-67.6)	0.021*
Xpert-	13	12					

Diagnostic results for RIF resistance

All the strains isolated were identified as Mycobacterium bovis. All strains were found to be susceptible to RIF in the GeneXpert method.

## Discussion

In Morocco, 90% of LNTBs are diagnosed based on biopsy and histopathological testing [14]. Our study has mainly assessed the performances of GeneXpert MTB/RIF in the diagnosis of LNTB and rifampicin resistance against the established bacteriological gold standard, using lymph node biopsies during surgical cervicectomy for patients with cervical adenopathy, in a prospective cohort of 75 patients admitted to the department of ENT in Hassan II Hospital at Fez. Secondly, it describes the profile of the patients included.

Biological performance

The positivity rate for GeneXpert on lymph node specimens was 66.7% (50/75 cases). This proportion is similar to other studies, with a positive rate in 61.5% of patients in Ethiopia [19] and 51% in India [20]. In comparison, it is higher than 47.3% of patients in a study from Morocco (2017) [14] and 46% of patients in a study from South Africa [21]. Using culture as the gold standard, we found that the sensitivity of the GeneXpert test for the diagnosis of LNTB was 78.6 %. It is lower than that reported by studies conducted in Tunisia (91.5%) [22], India (90%) [20], Ethiopia (87.8%) [19], and South Africa (80%) [21]. Although culture is generally used as a reference to validate the performance of new diagnostic tests, its major limitation is a prolonged turn-around time of 6-10 weeks [4].

Nevertheless, due to the paucibacillary character of the samples and the invasiveness of the procedure via lymph node biopsy for a positive diagnosis, histological examinations may support the diagnosis when there is a tuberculoid granuloma or giant cellular granuloma associated with caseous necrosis [17]. In our study, the specificity of histopathology in favor of LNTB was better than culture, when it showed a granuloma with caseous necrosis (52.5% vs. 40.4%). Our results are less significant than those reported by Bennani et al. (64.6%) [14]. The PPV of GeneXpert in our study was 44%, indicating that 56% of patients might be considered to have LNTB, whereas the GeneXpert test was negative. The NPV was 76% in the diagnosis of LNTB. Therefore, the proportion of false-negative cases would be 24%, which is lower than the results of studies from Tunisia (PPV: 100%; NPV: 92.7%) [22], and Morocco in 2017 (PPV: 74.1%%; NPV: 93.2%)[14].

Antibacillary resistance is one of the emerging issues in the management of TB. This is the root cause of the delay in diagnosis and treatment and an increase in deaths [6]. WHO estimates a rifampicin resistance rate of 3.3% in new cases and 17.7% in treated cases [23]. In our study, no resistance to rifampicin was found, while Mycobacterium bovis strains showed resistance to pyrazinamide in the study by Ghanian et al. [22]. Further studies are necessary to assess the performance of GeneXpert for rifampicin resistance detection.

Clinical characteristics

Milk-borne infection was the principal cause of cervical adenopathy, where Mycobacterium bovis was isolated in 12.5% of cases [24]. Despite the low prevalence of Mycobacterium bovis, their presence in the milk of animals is a public health concern, especially in patients from underdeveloped regions where consumption of contaminated raw milk is fairly common [25]. In our study, Lben was the milk type predominantly consumed (91.8%), followed by farm milk (60.7%). No statistical difference or association was found to be significant in this regard. In our study, most of the patients were young and female: 48% of them were between 15 and 29 years old, and 60% were females. These results align with the literature and can be attributed to the differences in the immune response between the two sexes [3,22,26].

Strengths and limitations

In our study, the GeneXpert demonstrated great sensitivity and NPV, making it the best screening test for LNTB. It can be useful to facilitate early detection and therefore treat and prevent infection, as a replacement test for the usual diagnosis (culture, histopathology). Our study has a few limitations, primarily the small sample size. Consequently, the findings cannot be generalized to the community at large.

## Conclusions

Based on our findings, the GeneXpert assay is an excellent alternative for the diagnosis of LNTB among patients with cervical adenopathy. There is a need to raise awareness so that GeneXpert is widely adopted throughout the country, and larger studies are needed in the future to validate our findings, especially in resource-limited countries.
